# Junctional adhesion molecule-A is overexpressed in advanced multiple myeloma and determines response to oncolytic reovirus

**DOI:** 10.18632/oncotarget.5753

**Published:** 2015-10-15

**Authors:** Kevin R. Kelly, Claudia M. Espitia, Weiguo Zhao, Erik Wendlandt, Guido Tricot, Fenghuang Zhan, Jennifer S. Carew, Steffan T. Nawrocki

**Affiliations:** ^1^ Anne Nohl Division of Hematology and Center for the Study of Blood Diseases, University of Southern California Keck School of Medicine, Los Angeles, CA, USA; ^2^ Department of Medicine and The Institute for Drug Development, Cancer Therapy and Research Center at The University of Texas Health Science Center, San Antonio, TX, USA; ^3^ Division of Hematology, Oncology, and Blood and Marrow Transplantation, Department of Internal Medicine, University of Iowa, Iowa City, IA, USA; ^4^ Translational Hematology and Oncology Research, Taussig Cancer Institute, Cleveland Clinic, Cleveland, OH, USA

**Keywords:** myeloma, reovirus, bortezomib, JAM-A, NOXA

## Abstract

Despite the development of several new agents for multiple myeloma (MM) therapy over the last decade, drug resistance continues to be a significant problem. Patients with relapsed/refractory disease have high mortality rates and desperately need new precision approaches that directly target specific molecular features that are prevalent in the refractory setting. Reolysin is a proprietary formulation of reovirus for cancer therapy that has demonstrated efficacy in multiple clinical trials. Its selective effects against solid tumors have been largely attributed to *RAS*-mediated control of reovirus replication. However, the mechanisms regulating its preferential anti-neoplastic effects in MM and other hematological malignancies have not been rigorously studied. Here we report that the reovirus receptor, junctional adhesion molecule-A (JAM-A) is highly expressed in primary cells from patients with MM and the majority of MM cell lines compared to normal controls. A series of experiments demonstrated that JAM-A expression, rather than RAS, was required for Reolysin-induced cell death in MM models. Notably, analysis of paired primary MM specimens revealed that JAM-A expression was significantly increased at relapse compared to diagnosis. Two different models of acquired resistance to bortezomib also displayed both higher JAM-A expression and elevated sensitivity to Reolysin compared to parental cells, suggesting that Reolysin may be an effective agent for patients with relapsed/refractory disease due to their high JAM-A levels. Taken together, these findings support further investigation of Reolysin for the treatment of patients with relapsed/refractory MM and of JAM-A as a predictive biomarker for sensitivity to Reolysin-induced cell death.

## INTRODUCTION

The outcome for patients with multiple myeloma (MM), a fatal neoplasm characterized by the uncontrolled proliferation of clonal plasma cells, has improved significantly due to the development of novel treatments such as proteasome inhibitors and immunomodulatory (IMiDs) agents [1, 2]. However, the disease remains incurable and patients that are refractory to both proteasome inhibitors and IMiDs have a particularly dismal prognosis [3]. MM cells produce large quantities of immunoglobulins and this heavy engagement in protein synthesis results in constitutive endoplasmic reticulum (ER) stress. Due to their high basal proteotoxic stress, MM cells are particularly vulnerable to agents that disrupt protein homeostasis [4–7].

Reovirus is a double-stranded RNA virus that is ubiquitous in the environment and typically causes asymptomatic infections of the respiratory and gastrointestinal tract of humans [8, 9]. The reovirus-based formulation Reolysin is being evaluated as an anticancer therapy on the basis of its selective replication in transformed cells [10–14]. We recently demonstrated that reovirus replication in MM cells induces a large accumulation of viral proteins that subsequently promotes ER stress-mediated apoptosis [12]. Combined treatment with bortezomib (BZ) and Reolysin yielded a dual accumulation of undegraded ubiquitinated proteins and viral particles resulting in the synergistic induction of ER stress and the BH3-only family member NOXA [12, 15]. These findings led to its investigation in a Phase 1 study in relapsed/refractory MM patients [16].

Despite a number of preclinical and clinical studies of the safety and efficacy of Reolysin, the mechanisms underlying its selectivity for malignant cells remain unclear. The ability of Reolysin to preferentially replicate in transformed cells has been attributed to increased RAS pathway activity that inhibits RNA-activated protein kinase (PKR) activation, thus allowing viral protein synthesis [17]. However, key studies have demonstrated that RAS activity does not always correlate with sensitivity to reovirus infection, which highlights that other factors are clearly important in some tumor types [18, 19].

Junctional adhesion molecule-A (JAM-A) is an immunoglobulin superfamily protein encoded by the *F11R* gene that is expressed on circulating neutrophils, monocytes, lymphocytes and platelets [20]. It has several diverse functions including the regulation of tight junctions between cells, leukocyte transmigration, differentiation of endothelial progenitor cells and platelet activation [21–24]. Roles for JAM-A as an important negative prognostic indicator in cancer and in the regulation of cancer progression and metastasis are beginning to emerge [25, 26].

JAM-A has also been shown to control the entry of reovirus into cells, but its specific role as a potential determinant of the sensitivity of malignant cells to Reolysin-induced cell death in cancer is not well defined [27, 28]. We investigated this in preclinical models of MM and primary patient specimens. Here we report that high JAM-A expression in MM cells is associated with reduced progression free survival and advanced disease and that sensitivity to Reolysin is at least partially dependent on JAM-A. In addition, acquired resistance to BZ leads to an induction in JAM-A expression that promotes enhanced sensitivity to Reolysin-induced cell death. Our data support our recently initiated Phase Ib study of Reolysin in combination with BZ for MM patients with relapsed/refractory disease.

## RESULTS

### Expression of the reovirus receptor JAM-A promotes reovirus replication and Reolysin-mediated apoptosis in MM cells

**Figure 1:**
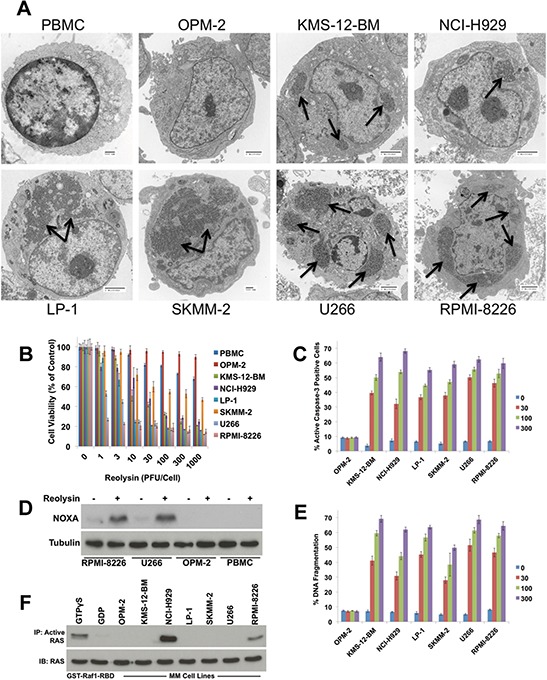
Reovirus replication in MM cells induces apoptosis independently of RAS activity status **A.** PBMCs from healthy donors and 7 MM cell lines were treated with 30 PFU/Cell Reolysin for 48 h. Reovirus replication was determined by transmission electron microscopy. Arrows denote reovirus accumulation. Bar represents 2 microns or 500 nm as indicated on each image. **B.** Reolysin decreases cell viability in MM cell lines while showing little activity against PBMCs or OPM-2 cells. PBMCs and MM cell lines were treated with the indicated amounts of Reolysin for 72 h and cell viability was measured by MTT assay. Mean ± SD, *n* = 3. **C.** Reolysin induces caspase-3 processing in all MM cell lines except OPM-2. MM cells were treated for 48 hours with the indicated concentrations of Reolysin. Active caspase-3 was measured using fluorescent antibody staining and flow cytometry. Mean ± SD, *n* = 3. **D.** Cells susceptible to Reolysin-mediated apoptosis induce NOXA expression. Cells were treated for 48 h with 30 PFU/Cell Reolysin. NOXA expression was determined by immunoblotting. **E.** Reolysin stimulates apoptosis in all MM cell lines except OPM-2. Cells were treated with the indicated concentrations of Reolysin and apoptosis was measured by PI-FACS analysis. Mean ± SD, *n* = 3. **F.** Determination of RAS activity in MM cell lines. Constitutively active RAS levels were determined in MM cell lines using an active RAS pull-down kit. GTPγS and GDP treated cells served as positive and negative controls, respectively.

Although Reolysin has been extensively investigated as an anti-cancer treatment, specific biomarkers that are predictive of clinical activity have not been validated. We hypothesized that JAM-A may regulate sensitivity to reovirus and that its expression could therefore be used to predict response to therapy. We first treated a panel of MM cell lines with Reolysin and assessed reovirus infection levels. Reolysin treatment was associated with significant intracellular viral accumulation in all lines evaluated except for OPM-2 cells, which like normal peripheral blood mononuclear cells (PBMC) did not exhibit detectable reovirus replication (Figure 1A). These results were consistent with the ability of Reolysin to reduce cell viability in that all MM cell lines showed a dose-dependent diminishment of viability with the exception of OPM-2 cells, which displayed a very minimal response to Reolysin that was similar to that of normal PBMCs from healthy donors (Figure 1B). Reolysin treatment also induced caspase-3 processing, an increase in NOXA expression, and DNA fragmentation in reovirus susceptible MM cell lines. However, OPM-2 and PBMCs remained largely unaffected by Reolysin treatment (Figures 1C, 1D, and 1E).

Previous reports have demonstrated that *RAS* mutated cancer cells are hypersensitive to reovirus infection and apoptosis [13, 17, 29–31]. Viral infection of normal cells activates PKR, which in turn phosphorylates eukaryotic initiation factor 2 α-subunit (eif2α) leading to inhibition of viral protein synthesis. In contrast, PKR activity is not stimulated in cells with an activated RAS pathway, which allows viral replication to continue in an unchecked manner [14, 17]. The relationship between activated RAS status and Reolysin sensitivity has been demonstrated in many solid tumor models. However, after performing DNA sequencing analyses on all of our MM cell lines, we were unable to establish a direct correlation between *RAS* mutation status and Reolysin sensitivity as multiple lines with wild-type *RAS* (e.g. U266 and LP-1) exhibited high sensitivity to Reolysin infection (Table 1). Since *RAS* mutation is only one mechanism that results in its constitutive activation, it is possible that evaluating RAS mutational status alone may be insufficient to determine the suitability of activated RAS as a predictor of Reolysin susceptibility. Considering this, we further evaluated *RAS* activity in MM cell lines using a RAS pull-down activation assay. These experiments revealed that the only MM cell lines displaying constitutive RAS activity were those that possessed *RAS* mutations (NCI-H929 and RPMI-8266) (Figure 1F), suggesting that other factors may regulate Reolysin sensitivity in MM.

**Table 1:** RAS mutation status in MM cell lines

Cell Line	N-RAS	K-RAS	nt Change
OPM-2	+	+	–
KMS-12-BM	+	+	–
NCI-H929	G13D	+	GGT > GAT
LP-1	+	+	–
SKMM-2	+	+	–
U266	+	+	–
RPMI-8226	+	G12A	GGT > GCT

+ : Wild-type

### JAM-A expression is elevated in Reolysin-sensitive MM cell lines and in patients with newly diagnosed MM and MGUS compared to normal cells

**Figure 2:**
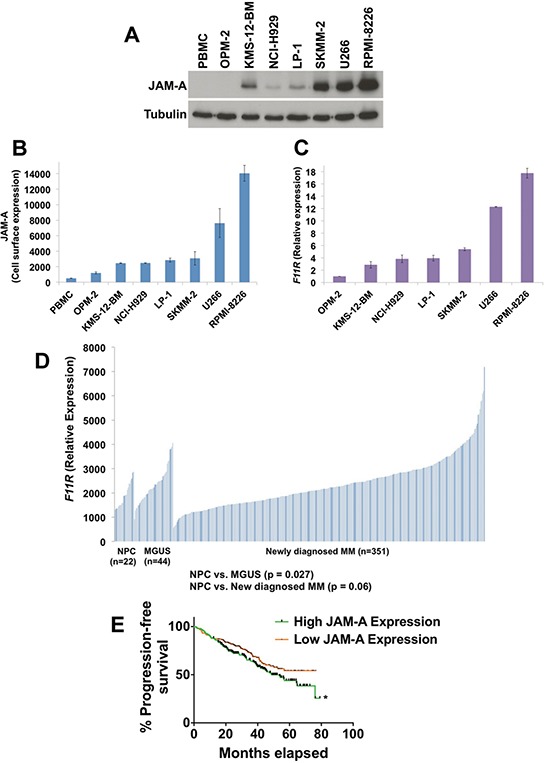
JAM-A is overexpressed in MM cells **A.** JAM-A was measured by immunoblotting in 7 MM cell lines and normal PBMCs. Tubulin served as a loading control. **B.** Cell surface expression of JAM-A on MM cell lines and PBMCs. JAM-A was measured by fluorescent staining and flow cytometry. Mean ± SD, *n* = 3. **C.** Measurement of JAM-A (*F11R*) transcript levels. *F11R* expression was determined in MM cell lines by qRT-PCR analysis. Mean ± SD, *n* = 3. **D.** *F11R* expression in normal plasma cells (NPC) from 22 healthy subjects, 44 subjects with MGUS, and 351 patients with newly diagnosed MM. **E.** Kaplan-Meier analyses of PFS revealed inferior outcomes among patients with high JAM-A expression compared with patients with low JAM-A expression. Groupings are based on quartiles with *p* values calculated for the top half compared to the bottom half (50/50) of JAM-A expression *Indicates a significant difference from low JAM-A expression group, *p* = 0.04.

Our electron microscopy analyses demonstrated a lack of intracellular reovirus particles in OPM-2 cells, suggesting that their resistance to Reolysin may be due to ineffective reovirus entry (Figure 1A). As mentioned earlier, JAM-A has previously been reported to be a receptor for reovirus cell attachment [27]. We hypothesized that differential expression of JAM-A may account for the heterogeneous sensitivity of MM cell lines to Reolysin. Consistent with this idea, measurement of JAM-A levels by immunoblotting, flow cytometry (cell surface expression), and qRT-PCR analysis revealed a correlation between JAM-A expression and Reolysin-induced cell death (Figures 2A, 2B, and 2C). Notably, the OPM-2 MM cell line expressed very low levels of JAM-A, suggesting that this may be the underlying cause of its resistance to Reolysin. The majority of the tested MM cell lines displayed significant JAM-A expression and were sensitive to reovirus infection and Reolysin-mediated apoptosis. We next evaluated the expression levels of JAM-A (*F11R*) in primary samples of normal plasma cells (NPC) (*n* = 22), MGUS (*n* = 44), and newly diagnosed MM (*n* = 351). Importantly *F11R* was significantly overexpressed in MGUS patients compared to NPC (*p* = 0.027) and trended toward significance in newly diagnosed MM patient samples compared to NPCs (*p* = 0.06) (Figure 2D). In addition, patients with high JAM-A expression had a shorter progression free survival compared to those with low expression by Kaplan-Meier analysis (*p* = 0.04) (Figure 2E). This data suggests that high *F11R* expression is associated with an inferior outcome in MM.

### JAM-A expression is a key determinant of sensitivity to Reolysin in MM cell lines

**Figure 3:**
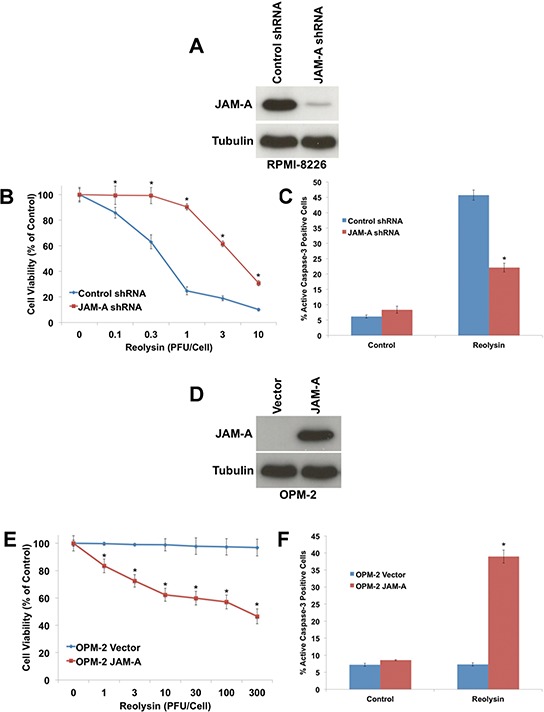
JAM-A regulates reovirus sensitivity in MM **A.** shRNA-mediated knockdown of JAM-A in RPMI-8226 cells. Cells were infected with JAM-A lentiviral shRNA and knockdown was determined by immunoblotting. **B.** Cells with silenced JAM-A are significantly less sensitive to Reolysin. Cells infected with control or JAM-A shRNA were treated with Reolysin for 72 h. Cell viability was measured by MTT assay. Mean ± SD, *n* = 3, *p < 0.05 compared to shRNA control treated cells. **C.** Cells with decreased JAM-A levels are resistant to Reolysin-mediated apoptosis. RPMI-8226 cells were treated with 30 PFU/Cell Reolysin for 48 h. Active caspase-3 levels were measured by fluorescent staining and flow cytometry. Mean ± SD, *n* = 3, *p < 0.05 compared to shRNA control treated cells. **D.** Overexpression of JAM-A in OPM-2 cells. JAM-A was transfected into OPM-2 cells and immunoblotting confirmed increased expression at 72 h following transfection. **E.** Forced expression of JAM-A sensitizes OPM-2 cells to Reolysin. Vector control and JAM-A transfected cells were treated with the indicated concentrations of Reolysin for 72 h. Cell viability was measured by MTT assay. Mean ± SD, *n* = 3, *p < 0.05 compared to Vector control treated cells. **F.** JAM-A overexpression sensitizes OPM-2 cells to Reolysin-mediated apoptosis. OPM-2 cells were treated with 30 PFU/Cell Reolysin for 48 h and apoptosis was measured by active caspase-3 staining and flow cytometry. Mean ± SD, *n* = 3, *p < 0.05 compared to Vector control treated cells.

To further investigate the role of JAM-A in Reolysin sensitivity, we used shRNA to knockdown its expression in RPMI-8226 cells, which exhibit high basal JAM-A levels (Figure 3A). Targeted stable knockdown of JAM-A rendered RPMI-8226 cells significantly less sensitive to Reolysin-mediated cell death as measured by cell viability analysis and caspase-3 activation (Figures 3B and 3C). Conversely, we also overexpressed JAM-A in the Reolysin-resistant OPM-2 cell line (Figure 3D). Importantly, overexpression of JAM-A was sufficient to sensitize the resistant OPM-2 cells to Reolysin as demonstrated by MTT and active caspase-3 assays (Figures 3E and 3F). Collectively, these data support a role for JAM-A expression as a key determinant of sensitivity to Reolysin in MM.

### JAM-A expression increases significantly during disease progression and correlates with increased sensitivity to Reolysin

**Figure 4:**
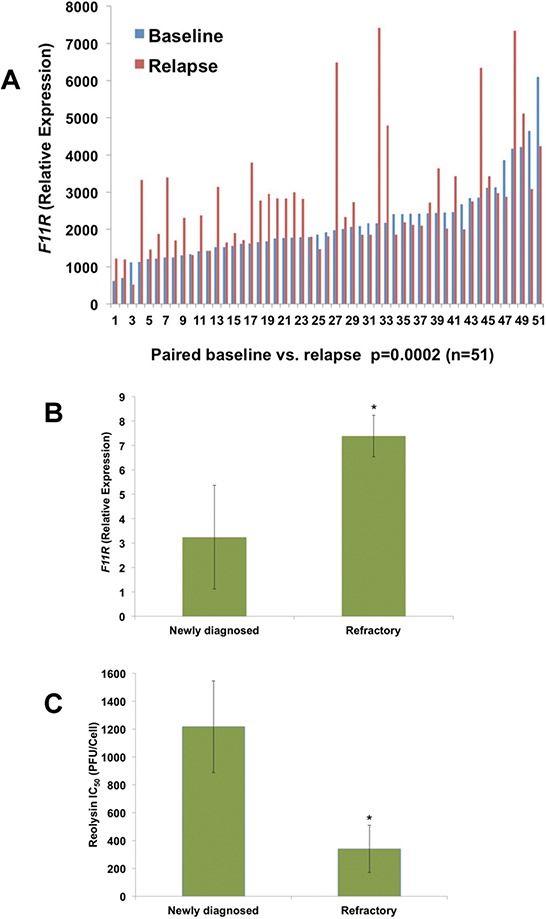
JAM-A (F11R) expression is significantly upregulated in MM patients at relapse **A.** *F11R* levels are increased in MM patients at relapse. A total of 51 paired CD138+ specimens from MM patients were measured for *F11R* expression (*P* = 0.0002). **B.** *F11R* levels are increased in freshly isolated CD138+ cells from refractory/relapsed MM patients (*n* = 4) and one refractory Waldenstrom's macroglobulinemia patient compared to those with newly diagnosed MM (*n* = 6). *F11R* was determined by qRT-PCR. Mean ± SD, *p < 0.05 compared to newly diagnosed MM. **C.** Relapsed/refractory MM/WM patients (*n* = 5) display increased sensitivity to Reolysin compared to newly diagnosed MM patients (*n* = 6). CD138+ cells from patients were treated with varying concentrations of Reolysin and cell viability was determined by MTT assay. IC_50_ values to Reolysin were calculated and plotted. Mean ± SD, *p < 0.05 compared to newly diagnosed MM cells treated with Reolysin.

Given that increased JAM-A (*F11R*) expression may be associated with inferior outcome in MM patients, we compared the gene expression profile (GEP) of 51 paired MM samples collected at baseline and at early relapse and found that *F11R* was significantly overexpressed at relapse compared to diagnosis (*p* = 0.0002) (Figure 4A). To further evaluate the relationship between elevated JAM-A expression and refractoriness, we measured *F11R* expression by qRT-PCR in fresh CD138+ cells isolated from 6 patients with newly diagnosed MM, 4 MM patients who were relapsed/refractory to BZ and lenalidomide, and 1 Waldenstrom's macroglobulinemia patient who was refractory to BZ treatment (Table 2). Consistent with our previous data, *F11R* expression was significantly higher in relapsed/refractory patients (Figure 4B) compared to those that were newly diagnosed. Furthermore, the cells taken from patients with relapsed/refractory disease were also more sensitive to Reolysin treatment as demonstrated by significantly lower IC_50_ values (Figure 4C). Taken together, these findings suggest that Reolysin treatment may be particularly effective for MM patients who are refractory to or relapse following BZ treatment.

**Table 2:** Patient characteristics

Patient #	Age	Diagnosis	Cytogenetics	Prior Tx	Comments
1	74	MM (IgA Kappa)	normal	newly diagnosed	
2	53	MM (Lambda light chain)	normal	newly diagnosed	
3	61	MM (IgG Kappa)	hyperdiploid	newly diagnosed	
4	76	MM (IgG Kappa)	normal	newly diagnosed	
5	44	MM (Kappa light chain)	normal	newly diagnosed	
6	56	MM (IgG Kappa)	hyperdiploid	newly diagnosed	
7	53	MM (IgG Lambda and IgA Kappa)	hyperdiploid	Velcade, Dexamethasone, Revlimid, clinical trials	Refractory
8	71	MM (IgG Kappa)	hyperdiploid	Velcade, Revlimid, auto tx	Relapsed
9	42	MM (Kappa light chain)	normal	Velcade, Revlimid, auto tx	Relapsed
10	56	MM (IgG Lambda)	Trisomy 17, 13 q	Velcade, Revlimid, Decadron, auto tx	Relapsed
11	58	WM	normal	Velcade, Rituximab	Refractory

### JAM-A expression is increased in MM cell lines with acquired BZ resistance

**Figure 5:**
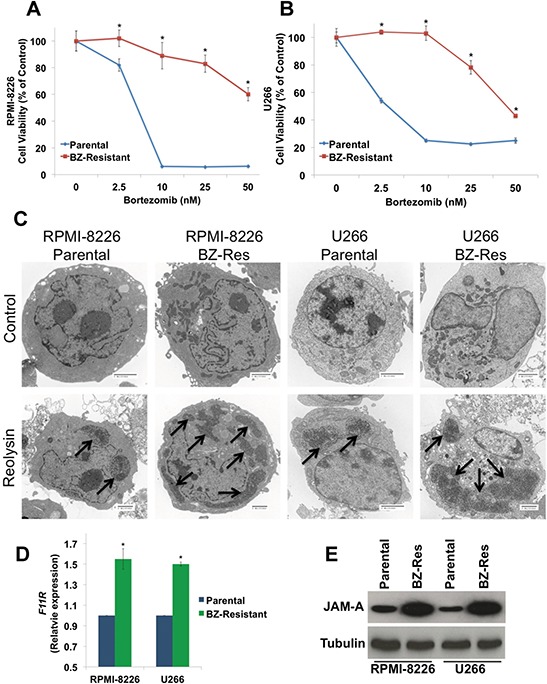
BZ-resistant MM cells display elevated JAM-A expression and enhanced reovirus replication **A-B**. BZ resistance in MM cell lines. Parental and BZ-resistant cells were treated with the indicated concentrations of BZ for 72 h. Cell viability was determined by MTT assay. Mean ± SD, *n* = 3. *p < 0.05 compared to parental cells treated with Reolysin. **C.** BZ-resistant cells display increased reovirus levels by electron microscopy. Cells were treated with 30 PFU/Cell Reolysin for 48 h and harvested for electron microscopy. Arrows denote reovirus accumulation. Bar represents 2 microns. **D-E**. BZ-resistant cells exhibit increased JAM-A expression. *F11R*/JAM-A levels were measured by qRT-PCR and immunoblotting. Mean ± SD, *n* = 3, *Significant increase compared to parental cells, *p* < 0.05.

To further assess the potential utility of Reolysin as a therapeutic option for MM patients with BZ resistance, we generated BZ-resistant RPMI-8226 and U266 MM cell lines by chronically exposing them to increasing concentrations of BZ over the span of one year. While 10 nM BZ (at 72 h) dramatically reduced cell viability in the parental RPMI-8226 and U266 cells, the corresponding resistant cell lines were relatively unaffected (Figures 5A and 5B). We next evaluated reovirus replication in the RPMI-8226 and U266 parental and BZ-resistant cells. Electron microscopy revealed an increase in reovirus products in the resistant cells compared to parental cells (Figure 5C). Consistent with increased reovirus products in the BZ-resistant cells and in agreement with our data generated in primary CD138+ cells isolated from MM patients, BZ-resistant cell lines displayed significantly higher levels of JAM-A expression compared to parental cells as measured by qRT-PCR and immunoblotting (Figures 5D and 5E).

### BZ-resistant cell lines are significantly more sensitive to Reolysin-mediated NOXA induction and apoptosis

**Figure 6:**
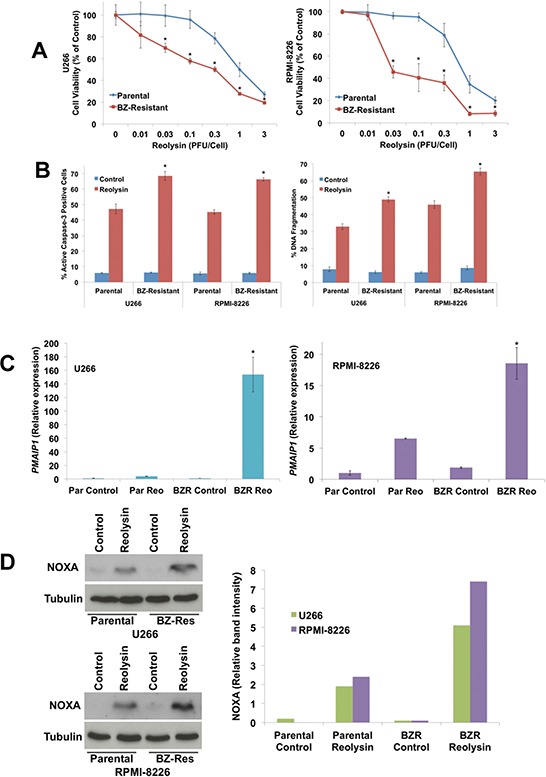
BZ-resistant MM cells exhibit increased sensitivity to Reolysin-induced NOXA upregulation and apoptosis **A.** BZ-resistant cells display increased sensitivity to Reolysin. Cells were treated with the indicated concentrations of Reolysin for 72 h. Cell viability was determined by MTT assay. Mean ± SD, *n* = 3, *Significant difference compared to Reolysin treated parental cells, *p* < 0.05. **B.** BZ-resistant cells are more sensitive to Reolysin-mediated apoptosis. Cells were treated with 30 PFU/Cell Reolysin for 48 h. Apoptosis was measured by active caspase-3 assay (Left) and PI-FACS analysis (Right). Mean ± SD, *n* = 3, *Significant increase compared to Reolysin treated parental cells, *p* < 0.05. **C-D**. NOXA (*PMAIP1*) expression following Reolysin treatment. Parental and BZ-resistant variants were treated with 30 PFU/Cell Reolysin for 48 h. *PMAIP1*/NOXA expression was measured by qRT-PCR and immunoblotting. Mean ± SD, *n* = 3, *Significant increase compared to Reolysin treated parental cells, *p* < 0.05. Immunoblot band intensity was quantified by densitometry.

We next investigated whether the increased viral accumulation in BZ-resistant MM cell lines would translate into heightened sensitivity to Reolysin-mediated cell death. Notably, Reolysin exposure induced significantly greater reductions in cell viability in the BZ-resistant cells versus the parental cell lines as measured by MTT assay (Figure 6A). The reduction in cell viability in the BZ-resistant cell lines following Reolysin treatment was associated with significantly greater levels of virus-induced apoptosis as measured by active caspase-3 induction and DNA fragmentation (Figure 6B). We have previously shown that the induction of NOXA is an important mediator of Reolysin-induced cell death in MM cells [12]. In order to determine whether enhanced NOXA induction contributed to the hypersensitivity in BZ-resistant variants, we quantified NOXA expression in both paired BZ-sensitive and -resistant MM cell lines compared to their parental counterparts by using qRT-PCR and immunoblotting. Indeed, Reolysin triggered significantly greater levels of NOXA expression in resistant compared to parental cells (Figures 6C and 6D). Collectively, our data suggest BZ resistance and disease progression is associated with increased JAM-A expression and this phenomenon may render such cells uniquely vulnerable to Reolysin therapy.

## DISCUSSION

Reolysin has undergone clinical evaluation in multiple Phase I and II trials for the treatment of various solid tumors and has demonstrated significant activity, while being safe and very well tolerated when administered locally or systemically [32–36]. However, it has not been rigorously investigated for the treatment of hematological malignancies. We and other investigators previously demonstrated that reovirus replication triggers ER stress and apoptosis in MM cells, resulting in significant activity against MM cell lines, primary cells from patients, and in animal models of MM [12, 37]. Furthermore, a recent study determined that reovirus was directly cytotoxic against chronic lymphocytic leukemia (CLL) cells [38]. Collectively, these data demonstrate that reovirus may have significant activity against hematological malignancies. In order to determine which MM patients may benefit most from this therapy, we sought to identify potential biomarkers of Reolysin sensitivity. While the mechanisms underlying the anticancer effects of Reolysin remain to be fully elucidated, previous studies have reported that reovirus selectively replicates in cells with an activated RAS pathway [14, 17]. We have also observed the importance of the relationship between RAS mutation/activity and reovirus infectivity in pancreatic cancer models [15]. However, in this study we did not observe a correlation between RAS activity and the sensitivity of MM cells to Reolysin. This indicated that other RAS-independent factors may regulate reovirus susceptibility in malignant plasma cells. Our observations are in agreement with recent investigations, which have also shown that the vulnerability of malignant cells to reovirus infection may not be dependent upon *RAS* mutation or an activated RAS pathway (e.g. constitutive EGFR activation) [18, 19]. This emerging data suggests that the specific factors regulating the susceptibility of malignant cells to reovirus infection are likely cell-type dependent.

JAM-A, a member of the immunoglobulin superfamily and important regulator of tight junction assembly, was previously identified as a cell surface receptor for reovirus attachment that facilitates endocytosis-mediated reovirus internalization [27]. However, its potential role in the control of the sensitivity of malignant cells to reovirus therapy has not been well studied, We became highly interested in specifically investigating JAM-A's contribution to this phenomenon after observing deficient reovirus cell entry in the OPM-2 MM cell line and hypothesized that their extremely low levels of JAM-A prevented reovirus internalization even after exposure to very high reovirus titers. Our data supported this hypothesis as we demonstrated that overexpression of JAM-A was sufficient to mediate reovirus infection and Reolysin-mediated cell death in OPM-2 cells. Accordingly, the reciprocal targeted JAM-A knockdown experiments showed that reducing JAM-A levels severely diminished the ability of MM cells to die in response to Reolysin treatment.

Reolysin displayed significant, but heterogeneous activity against the majority of MM cell lines and MM primary patient specimens tested. Notably, there was a correlation between high JAM-A levels and increased sensitivity. To better understand the context of JAM-A expression in MM pathogenesis, we evaluated its levels in NPC, MGUS, and newly diagnosed MM patients. JAM-A expression was elevated in patients with MGUS and newly diagnosed MM compared to NPC, suggesting a potential role for JAM-A during malignant transformation. These results provide rationale for further evaluating the potential efficacy of Reolysin for specific tumor types that exhibit high JAM-A expression.

While the use of proteasome inhibitors and IMiDs has improved MM patient care, primary refractory disease and acquired drug resistance are still significant clinical problems. Interestingly, in our study higher JAM-A expression was detected in MM patients at relapse or who were refractory to standard of care (BZ and lenalidomide) and was associated with inferior outcome. These data suggest that JAM-A itself may play a previously unknown role during the evolution of drug resistance. Indeed, our results are congruent with other reports of studies in nasopharyngeal cancer, glioblastoma, non-small cell lung cancer, and breast cancer showing that elevated JAM-A expression is associated with metastasis, tumor progression, and poor prognosis [25, 26, 39–44]. Moreover, the increased JAM-A levels that we identified in relapsed/refractory MM cells may represent an “Achilles' Heel” that can specifically be therapeutically exploited by Reolysin treatment. Our data also define JAM-A as a candidate predictive biomarker of Reolysin sensitivity that can be further evaluated in the clinical setting.

New therapeutic approaches are desperately needed for MM patients who relapse or are refractory to BZ therapy. Initial clinical investigation of Reolysin as a monotherapy for MM revealed that it was well tolerated and suitable for testing in combination with conventional chemotherapy [16]. We are currently conducting a hypothesis-driven investigator-initiated Phase Ib clinical trial (NCT02514382) of Reolysin in combination with BZ and dexamethasone in patients with relapsed/refractory MM. Based on our preclinical data, we are evaluating baseline JAM-A expression levels and pharmacodynamic induction of the ER stress apoptotic response as potential biomarkers of Reolysin sensitivity in primary specimens from patients treated on this trial. We anticipate that our collective preclinical and clinical findings will provide significant new insights regarding strategies to optimize the efficacy of Reolysin therapy for patients with relapsed/refractory MM and other malignancies.

## MATERIALS AND METHODS

### Cells and cell culture

RPMI-8226, U266, and NCI-H929 MM cell lines were obtained from American Type Cell Culture Collection (Manassas, VA). LP-1, KMS-12-BM, SKMM-2, and OPM-2 were purchased from the DSMZ (Braunschweig, Germany). MM cell lines were maintained in RPMI-1640 media supplemented with 10% fetal bovine serum in a humidified incubator 37(C with 5% CO_2_. To establish human MM cells resistant to BZ, RPMI-8226 and U266 cells were continuously cultured in gradually increasing concentrations of BZ (initially 1 nM and increasing in increments of 1 nM over one year) to 50 nM (RPMI-8226) and 20 nM (U266), respectively. Normal peripheral blood mononuclear cells (PBMCs) were purchased from Stemcell Technologies (Vancouver, BC). Primary human MM cells were obtained from the bone marrow of MM patients at the CTRC/UTHSCSA after obtaining informed consent in accordance with an approved IRB protocol. CD138+ cells were selected using beads from Miltenyi Biotec (Auburn, CA).

### Antibodies and reagents

Antibodies were obtained from the following commercial sources: anti-NOXA (Calbiochem, Gibbstown, NJ), anti-tubulin (Sigma-Aldrich, St. Louis, MO) and anti-JAM-A (Santa Cruz Biotechnology, Santa Cruz, CA for flow cytometry, abcam, Cambridge, MA for immunoblotting). Reolysin was provided by Oncolytics Biotech Inc. (Calgary, AB). Bortezomib (VelcadeTM) was obtained from Millennium Pharmaceuticals.

### Transmission electron microscopy

MM cells and PBMCs were treated with 30 PFU/Cell Reolysin for 48 h and processed for electron microscopy. Sections were cut in an LKB Ultracut microtome (Leica, Deerfield, IL), stained with uranyl acetate and lead citrate, and examined in a JEM 1230 transmission electron microscope (JEOL, USA, Inc., Peabody, MA).

### Quantification of drug-induced cytotoxicity

Cell viability was assessed by 3-(4,5-dimethylthiazol-2-yl)-2,5-diphenyltetrazolium bromide (MTT) assay. Cells were cultured in 96-well plates at a density of 10,000 cells per well and were treated with Reolysin or BZ for 72 h. Following drug treatment, MTT was added and viability was quantified using a microplate reader. The pro-apoptotic effects of Reolysin and BZ were quantified by propidium iodide (PI) staining and fluorescence activated cell sorting (FACS) analysis of sub-G_0_/G_1_ DNA and quantification of active caspase-3 positive cells by flow cytometry using a commercial kit (BD Biosciences, San Jose, CA) as previously described [4].

### Immunoblotting

MM cells were lysed as previously described [6]. Protein from each sample was subjected to SDS-PAGE. Proteins were subsequently transferred to nitrocellulose membranes and blocked with 5% milk in a Tris-buffered saline solution containing 0.1% Tween-20 for 1 h. The blots were probed overnight with primary antibodies, washed, and then probed with species-specific secondary antibodies coupled to HRP. Immunoreactive material was detected by enhanced chemiluminescence (Alpha Innotech, San Jose, CA).

### RAS pull-down activation assay

RAS activity was determined using the active RAS pull-down and detection kit (Millipore, Rockford, IL) according to the manufacturer's instructions. Bound proteins were then subjected to SDS-PAGE and probed with an anti-RAS antibody. Whole lysates were run to confirm equal loading.

### Quantification of cell surface JAM-A levels by flow cytometry

MM cell lines and PBMCs were harvested and washed once in PBS, resuspended in FACS buffer (PBS, 5% fetal bovine serum, and 0.01% NaN_3_), and stained with an anti-JAM-A antibody (Santa Cruz Biotechnology, Santa Cruz, CA) followed by an Alexa Fluor 488 secondary antibody (Molecular Probes, Grand Island, NY). Following staining, cells were washed twice in FACS buffer and then resuspended in 500 μL of FACS buffer for measurement of the intensity of JAM-A fluorescence by flow cytometry (BD FACS Canto II, BD Biosciences, San Jose, CA).

### Quantitative real time polymerase chain reaction (qRT-PCR)

cDNA from untreated controls or Reolysin-treated cells were used for relative quantification by RT–PCR analyses. First-strand cDNA synthesis was performed from 1 μg RNA in a 20 μl reaction mixture using the high-capacity cDNA Reverse Transcription Kit (Applied Biosystems, Grand Island, NY) *F11R* and *PMAIP1* transcripts were amplified using commercially available TaqMan^®^ Gene expression assays (Applied Biosystems). *GAPDH* was used as a housekeeping gene.

### JAM-A expression profiling using the Affymetrix U133Plus2.0 microarray

Gene expression profiling data under accession number GSE2658 were collected from a publicly available website that includes 351 newly diagnosed patients with MM who participated in the Total Therapy 2 (TT2) clinical trial, 44 patients with monoclonal gammopathy of unknown significance (MGUS), and 22 normal plasma cell samples that were obtained from healthy donors [45, 46].

### shRNA knockdown of JAM-A

MM cells were infected with a lentivirus encoding a short hairpin RNA (shRNA) sequence specific for JAM-A or an empty vector (Santa Cruz Biotechnology, Santa Cruz, CA). Infected cells were selected with constant culture in 1 μg/ml puromycin. JAM-A knockdown was confirmed by immunoblotting.

### Overexpression of JAM-A

*F11R* overexpression was carried out using the pCMV6-hF11R expression vector (Origene, Rockville, MD). Plasmids were isolated using a Qiagen mini-prep plasmid isolation kit (Qiagen Inc., Valencia, CA). OPM-2 cells were transiently transfected with pCMV6-*hF11R* and the empty vector pCMV6 using the Transfast reagent (Promega, Madison, WI) according to the manufacturer's instructions. Briefly, 1 μg of each plasmid was diluted in serum-free medium. The diluted plasmids were vortexed before the addition of 3 μl Transfast reagent to bring the Transfast/DNA ratio to 1:1. Cells in Transfast/DNA mixture were then incubated at 37°C for 1 h. Positively transfected cells were subsequently selected using puromycin.

### RAS sequencing

DNA from MM cells was isolated using the DNeasy mini kit (Qiagen Inc., Valencia, CA). DNA was eluted with 100 μl nuclease-free water and samples were checked for concentration and quality using a NanoDrop spectrophotometer. PCR amplifications were conducted using optimized cycling conditions per gene-exon. All samples were sequenced with forward and reverse primers spanning all exons to obtain the complete sequence of each gene. Sequencing reactions were run on an ABI 3130xl at the Nucleic Acid Core Facility at the University of Texas Health Science Center.

### Statistical analyses

The Student's *t* test was used to compare two experimental groups. The correlation between JAM-A expression and disease progression was measured using the Kaplan-Meier method, and the log-rank test was used for group comparison. Significance was set at *p* < 0.05.
